# Not so Fast: Co-Requirements for Sonic Hedgehog Induced Brain Tumorigenesis

**DOI:** 10.3390/cancers7030848

**Published:** 2015-08-06

**Authors:** Stacey A. Ward, Joshua B. Rubin

**Affiliations:** Department of Pediatrics, Division of Hematology/Oncology, Washington University in St. Louis School of Medicine, Campus Box 8208, 660 South Euclid Ave, St. Louis, MO 63110, USA; E-Mail: ward_s@kids.wustl.edu

**Keywords:** Sonic hedgehog, cerebellum, medulloblastoma, GPCR, signal transduction

## Abstract

The Sonic hedgehog (Shh) pathway plays an integral role in cellular proliferation during normal brain development and also drives growth in a variety of cancers including brain cancer. Clinical trials of Shh pathway inhibitors for brain tumors have yielded disappointing results, indicating a more nuanced role for Shh signaling. We postulate that Shh signaling does not work alone but requires co-activation of other signaling pathways for tumorigenesis and stem cell maintenance. This review will focus on the interplay between the Shh pathway and these pathways to promote tumor growth in brain tumors, presenting opportunities for the study of combinatorial therapies.

## 1. Introduction

Medulloblastoma is the most common malignant pediatric brain cancer, with approximately 500 new diagnoses each year in the US [1]. This tumor occurs in the posterior fossa of the brain, primarily involving the cerebellum and adjacent brain regions. Medulloblastoma are WHO grade IV tumors (malignant and invasive) and are frequently categorized based on their histological staining as classic (sheets of small, round, blue cells), desmoplastic/nodular (nodes of reticulin-free areas surrounded by densely packed, highly proliferative cells), or large cell/anaplastic (widespread areas of nuclear polymorphism and atypia and a large cell appearance) [2]. However, histological subtyping alone has had limited utility for treatment stratification or prognostication, as patients within each histological subtype respond well, adequately or poorly to current therapies. The field has embraced technological advances including genomic sequencing and gene expression profiling to subtype medulloblastoma tumors into four groups—Wnt, Sonic hedgehog (Shh), Group 3/C-myc, and Group 4, based on genetic mutation and signaling pathway gene expression [3,4]. It has become clear that there is prognostic significance in separating tumors this way, since Wnt tumors respond very well to therapy (and reductions in therapy are currently being evaluating in clinical trials), while Group 3 and Group 4 tumors respond very poorly to therapy. Shh tumors exhibit intermediate response to current regimens, with clear outcome contributions of age at presentation and metastatic load contributing to tumor recurrence [1]. In this way, we have made great strides in separating tumors according to their oncogenic drivers and have succeeded in identifying druggable targets that are currently being investigated.

Shh medulloblastomas are the best-characterized form of the disease, as productive parallels have been drawn to granule neuron progenitor (GNP) expansion during normal cerebellar development, and multiple mouse models of tumorigenesis are available [5,6,7]. Cerebellar development primarily occurs post-birth, during which time GNPs proliferate and subsequently differentiate into granule neurons to make up the single most abundant neuronal cell type in the brain. *In vivo* and *in vitro* studies have definitively demonstrated that GNP proliferation is primarily regulated by Purkinje cell-derived Shh [8]. In the absence of Shh ligand, GNPs do not proliferate and migrate early from the external granule cell layer (EGL) of the developing cerebellum into the internal granule cell layer (IGL) while differentiating into granule neurons, resulting in a small cerebellum. A convergence of signals acts to retain GNPs in the EGL during proliferation, including CXCL12 and BDNF [9,10]. Although Purkinje cells secrete Shh well past the time of cerebellar development, GNPs only respond to this signal with a proliferative response during a discrete time, indicating that other pathways must contribute to maintenance of division as well as the promotion of migration and differentiation. However, the strong mitogenic response of these cells to Shh also predisposes them to abnormal proliferation in the setting of mutational activation of the Shh pathway.

It was observed that patients with Gorlin syndrome are predisposed to multiple cancers, including basal cell carcinoma and medulloblastoma [11]. It was further established that these patients carried a mutation in the *PTCH1* gene, resulting in constitutive activation of the Shh pathway, which drives growth of these tumors. Mouse models with similar mutations in the *PTCH1* gene also develop medulloblastomas histologically similar to human tumors [7]. Whole exome sequencing has identified other mutations in the Shh pathway found in human Shh tumors, including mutations in Patched (*PTCH*), Smoothened (*SMO*), Suppressor of Fused (*SuFu*), Gli family proteins, and *N-myc* [4,12]. These data indicate a clear role for the Shh signaling pathway in both normal cerebellar development and medulloblastoma growth but does not shed light on differences between normal and tumor cells.

The link between molecular subgroup and prognostic value has established that each tumor type will respond differently to treatment, in addition to identifying potential tumor driver mutations and pathways. This has led to the testing of multiple targeted therapies for medulloblastoma, specifically within the Shh subgroup. Most commonly, these clinical inhibitors target the Smoothened protein [13,14]. The most advanced of these treatments is the Genentech compound GDC-0449 (Vismodegib/Erivedge^®^), which directly binds to the Smo protein on the cell surface and blocks signaling downstream through repression of Gli activity and transactivation [15]. An initial Phase 1 trial included one patient with metastatic medulloblastoma who responded well to treatment, exhibiting tumor regression and improvement of quality of life [16]. However, after two months, his tumors recurred and he quickly succumbed to his disease. It was discovered that the recurrent tumors contained a mutation in the *Smo* gene, which made the protein refractory to GDC-0449 inhibition [17]. Whether this was the emergence of a new drug-resistant clone or selective deletion of only the sensitive (lacking mutational resistance) clones is unknown. Circumventing resistance to Shh antagonists will require understanding how it interacts with the multiple other growth-promoting pathways that are active during normal cerebellar development and in medulloblastoma. This review will focus on the interplay between the Shh and other pathways activated in medulloblastoma to promote growth and tumorigenesis, illuminating potential mechanisms of resistance and areas for further research into targeted therapies.

## 2. Shh Signaling

Shh is a secreted ligand that controls the development of various organs including the brain. Formation of a Shh gradient results in differential effects on target cells during embryogenesis. In the absence of ligand, the Shh receptor Ptch represses the activity of Smo, while binding of Shh to Ptch relieves this blockade. Smo is then free to move into the primary cilia, where it signals through Gli proteins to regulate gene expression [18]. There are three Gli family members expressed in vertebrates; Gli1 and Gli2 are mainly transactivators while Gli3 is a transcriptional repressor. Gli1 and 2 are maintained as full-length, active proteins in the presence of ligand, while Gli2 and Gli3 are cleaved into their repressive forms in the absence of ligand via PKA phosphorylation and subsequent ubiquitination [19,20]. Gli activation promotes the expression of a number of growth-promoting genes, including N-myc and pro-proliferative genes such as cyclin family members [21].

Mutational activation in the Shh pathway is common in multiple cancers, most notably basal cell carcinoma of the skin and medulloblastoma. Shh-subgroup medulloblastoma contain mutations in *Ptch*, *Smo*, and *SuFu*, as well as gene amplification of *Gli* family members and *N-myc* [4,12]. All of these mutations allow ligand-independent Shh signaling, promote cell proliferation, and increased tumorigenesis. In other cancers, including glioblastoma, there is absence of these mutations and evidence for ligand dependent Shh pathway activation [22]. The dispersal of Shh ligand and the magnitude of its effects are modulated by a complex array of post-translational events including pre-protein proteolytic processing [23], cholesterol modification of the carboxy terminus and palmitoylation of the amino terminus [24], oligomerization of the secreted protein, interaction between the oligomers and cell surface heparan sulfate proteoglycans [25] and further protoeolytic processing that results in Shh release and competency for Ptch binding [26].

As there is a clear link between activation of the Shh pathway (either through mutation or ligand-dependent signaling), cell growth and tumorigenesis, therapeutic blockade of Shh signaling has been pursued as a cancer treatment. Multiple inhibitors of Shh signaling have been developed, primarily targeting Smo activity (cyclopamine, GDC-0449 Genentech, LDE225 and LEQ506 Novartis, *etc.*) [27]. There are currently nine trials evaluating Shh inhibition in the treatment of pediatric or adult medulloblastoma, either alone or in combination with chemotherapy or radiation [28]. However, published studies indicate that single agent therapy will not be adequate to block tumor recurrence and growth, due to the emergence of drug-resistant clones [16,17]. In some cases, drug resistance is associated with the emergence of clones possessing mutations in drug binding sites [17]. However, it is not clear that this is the only mechanism of resistance. Studies of Shh signaling and function in multiple tissues (cerebellum, bone marrow) have highlighted requirements for co-activity of other pathways. Therefore, there is an immediate need to further define the molecular bases for these pathway interactions and determine whether they can promote resistance to Shh blockade and whether they represent opportunities for co-targeting in cancer treatment.

## 3. Wnt Signaling

The family of Wnt proteins contains secreted ligands which bind to Frizzled G protein coupled receptors (GPCRs) and affiliated coreceptors in the plasma membrane [29]. Canonical Wnt signaling is initiated by ligand binding, which results in stabilization of the β-catenin protein. Stabilized β-catenin then translocates into the nucleus and promotes proliferation through increased gene expression of proliferative genes such as cyclin D1 and c-myc. The noncanonical Wnt signaling pathway is independent of β-catenin and regulates cell shape and intracellular calcium levels [30].

Wnt pathway activation is recognized to be integral to tumor biology in the Wnt subgroup of medulloblastoma, primary through mutational activation of the β-catenin gene and active signaling in the absence of ligand [12]. Patients with a Wnt subgroup tumor have the best prognosis of the four tumor types. However, there is evidence that the Wnt pathway may play a role in Shh signaling in the Shh subgroup of tumors as well. For example, expression profiling of human medulloblastoma samples indicates that Wnt tumors can overexpress N-myc (a marker for Shh driven tumors), and Shh tumors are enriched in Wnt pathway genes [3]. In addition, both Wnt and Shh driven tumors express genes implicated in axonal guidance, suggesting a common signaling pathway convergence point [3]. Many β-catenin target genes overlap with Gli target genes, including N-myc, cyclin D, and NeuroD1 [31]. It is likely that these pathways share common signaling modulators, yet the specifics of this remain unclear.

## 4. Notch Signaling

Binding of the transmembrane ligands Delta-like and Jagged to members of the Notch receptor family results in gamma-secretase-mediated cleavage of the receptor and nuclear translocation of the intracellular domain and subsequent activation of gene expression (such as HES-1) [32]. In addition, the ligand-bound extracellular portion of Notch is cleaved and endocytosed by the ligand-expressing cell, possibly initiating autocrine signaling. Notch signaling promotes proliferation during neurogenesis, and inhibition of signaling results in progenitor cell differentiation [33,34]. During normal cerebellar development, Notch2 is required for proliferation and maintenance of a stem-like fate of GNPs [35], suggesting an additive role in Shh-induced proliferation.

Regardless of molecular subgroup, human medulloblastoma samples exhibit increased expression of JAG-1, DLL-1, Hes1 and Notch2 and decreased expression of JAG-2 and DLL-4 as compared to normal cerebellum [36]. In addition, human tumors expressing high levels of HES1 possess a poor prognosis, indicating that active Notch signaling is detrimental to patient survival [37]. Knocking down expression of JAG1 in Daoy cells, a Ptch-mutant medulloblastoma cell line, resulted in a decrease in proliferation and an increase in cleaved caspase 3/7, indicators of apoptosis [38]. Notch signaling has also been shown to regulate stemness, whereby inhibition of the pathway using MiR-34a in Daoy cells resulted in a decrease in the CD133+/CD15+ fraction of cultured cells [39]. Thus, it is possible that Shh activity drives proliferation while Shh-activation of Notch signaling promotes stemness, facilitating tumor growth and evolution.

Shh pathway activation also feeds forward on the Notch pathway, since Smo activation causes the transactivation of the *Hes1* gene in a Notch-ligand independent fashion as well as expression of JAG2 [40]. Shh activation through heterozygous inactivation of Ptch resulted in an increase in gene expression downstream of Notch activation [41]. In a mouse model of medulloblastoma which overexpresses the activated form of intracellular Notch1 and decreased levels of p53, mice develop medulloblastoma tumors which closely mimic the Shh-subgroup of tumors, indicating that each pathway can crosstalk with the other, although Notch pathway activation is not necessary for the growth of Shh tumors [42,43,44]. Thus, Notch activation is downstream of Smo activation, and represents a potential targetable pathway in the treatment of Shh-medulloblastoma.

## 5. BMP Signaling

Bone morphogenetic proteins (BMPs) are secreted ligands first implicated in the growth of bone and cartilage. It is now recognized that BMP signaling is important in the development of multiple organs, including the brain [45]. BMP ligands bind to their receptors (BMPRs) and activate signaling through SMAD proteins, primarily acting through translocation to the nucleus and transactivation of gene expression [46].

BMPs are inhibitory to Shh signaling in the cerebellum. They are required to differentiate neural progenitors into cells in the GNP pathway [47], but prolonged signaling will inhibit Shh-induced proliferation of GNPs [48]. Overexpression of the BMP effector SMAD5 is sufficient to induce differentiation of GNPs into granule neurons [48]. Thus, although BMP signaling is required for lineage specification, it is also capable of blocking Shh-induced proliferation in the normal cerebellum.

BMPs can also inhibit proliferation of Shh-MB without inducing apoptosis, as shown using a mouse model of medulloblastoma [49]. The mechanism of action was determined to be through downregulation of *Atoh1*/Math1 expression, a bHLH transcription factor that is important for granule lineage specification and Sonic hedgehog responsiveness. The addition of BMP to medulloblastoma cell cultures also induced apoptosis [50]. Therefore, activation of BMP effectors may represent a targetable pathway in addition to inhibition of Shh signaling.

## 6. bFGF Signaling

Fibroblast growth factors (FGF) are secreted ligands which play roles in angiogenesis, wound healing, and organ development [46]. FGF2 is also known as basic FGF (bFGF), as it will be referred to here. FGFs bind heparan sulfate moieties on the cell surface, in conjunction with FGF receptor family members.

It has been shown that the addition of bFGF to purified GNP cultures inhibits cell proliferation even in the presence of Shh ligand, indicating a strong inhibitory effect downstream of Shh [51,52,53]. In fact, bFGF treatment blocks expression of Shh target genes in the presence of Shh ligand, and this requires the action of FGF receptors and MAPK activity [52]. The inhibition of Shh and downstream bFGF signaling results in increased differentiation of GNPs in culture [52].

BFGF also inhibits downstream signaling and proliferation in Shh-subgroup medulloblastoma cells [52,53]. This blockade was downstream of FGF receptors, upstream of Gli activation and required active MAPK signaling [53]. Tumorigenesis involved little to no bFGF signaling, as treatment of medulloblastoma cells with bFGF in culture decreased their tumor implantation rate *in vivo* while bFGF treatment of mice bearing medulloblastoma tumors blocked tumor growth [53]. These data indicate that bFGF inhibition of Shh signaling is downstream of the Shh ligand/receptor interaction, and represents a promising avenue for treatment of Smo-inhibitor resistant tumors.

## 7. PTEN/Akt/PIK3/PKB Signaling

Phosphatase and tensin homolog (PTEN) is an intracellular protein involved in signal transduction. It is classified as a tumor suppressor, is mutated in a multitude of tumor types and is responsible for the dephosphorylation of phosphoinositol in the plasma membrane to negatively regulate levels of PIP3 [54,55]. It also negatively regulates Akt/PIK3/PKB signaling [56].

Loss of the PTEN locus is a common genetic event in medulloblastoma, including an association with the Shh subgroup [57,58]. PTEN loss accelerates tumor progression in mouse models of Shh medulloblastoma, but is not required for tumor formation [59,60]. Loss of PTEN and coordinate activation of Akt is correlated with increased proliferative index of primary human medulloblastoma tumors [60,61]. PTEN loss promotes differentiation and blocks apoptosis of tumor cells *in vivo*, as well as downregulation of Shh pathway targets [60]. Low PTEN expression is also correlated with decreased survival in human patients [60]. Since loss of PTEN activates Akt/PIK3/PKB signaling and promotes tumor formation, these downstream pathways represent targetable areas for co-inhibition with Shh pathway inhibitors for the treatment of medulloblastoma.

## 8. CXCR4 Signaling

G-protein coupled receptors comprise a family of seven-pass transmembrane receptors responsible for sensing extracellular signals and transducing them to the intracellular environment [62]. Approximately 50% of current pharmacologicals target some of the 850 GPCR family members [63], making this class of proteins a widely studied and promising avenue of study for tumor treatment. CXCR4 is a Gi-coupled GPCR highly expressed during normal brain development on neurons and glia, but its expression decreases with time. However, it has been shown to be highly expressed in multiple forms of brain tumors, including medulloblastoma [64]. We showed a requirement for CXCR4 signaling in medulloblastoma xenografts using a clinically approved inhibitor, AMD3100 (Plerixafor/Mozobil®, Genzyme) which blocked *in vivo* growth of tumors [64]. CXCR4 binds to its ligand, CXCL12/SDF-1 (expressed by endothelial cells) and signals through the heterotrimeric G protein Gi to promote calcium mobilization, decreased cAMP levels, and PI3K and MAPK protein phosphorylation [65]. However, this pathway contains a built-in safety mechanism termed desensitization, whereby ligand binding also activates CXCR4 phosphorylation by G-protein coupled receptor kinases (GRKs), promoting its internalization via arrestin and clathrin recruitment and recycling via endosome.

CXCR4 has been shown to play a role in cerebellar development, with the highest level of expression found during the peak of GNP proliferation [9,66]. CXCR4 is known to regulate GNP migration as well, through the action of a protein phosphatase [67]. However, ligand-induced proliferation required concurrent Shh signaling, indicating that these pathways must act in concert to promote cell growth [9]. Shh activation is required for ligand-induced Gαq activation in cultured GNPs, since pre-treatment of cells with Shh was required for calcium mobilization [67].

Shh-driven medulloblastoma also exhibits the highest levels of CXCR4 gene expression [64], indicating that Shh-induced tumorigenesis may upregulate CXCR4 expression. In fact, we have shown that CXCR4 inhibition has an anti-proliferative effect on Shh-driven medulloblastoma tumors *in vivo*, through downmodulation of cyclin D1 expression [64]. Since CXCR4 inhibitors are currently in use for the treatment of multiple myeloma and lymphoma, and are in clinical trial for the treatment of glioblastoma and other brain tumors (NCT01977677, NCT01339039), their clinical evaluation for the treatment of medulloblastoma is feasible and represents an area for further investigation.

## 9. cAMP Signaling

GPCRs, such as CXCR4, frequently act through 3′, 5′-cyclic adenosine monophosphate (cAMP). Ligand-bound GPCRs function as guanine nucleotide exchange factors for the alpha subunits of heterotrimeric G proteins and promote their exchange of GDP for GTP and activation of heterotrimeric G protein signaling. There are four families of heterotrimeric G-proteins defined by the functions of their alpha subunits. Gαi and Gαs-containing G proteins regulate cAMP levels within a cell by inhibiting and activating its synthesis by adenylate cyclase, respectively [68,69].

High cAMP levels are known to block Shh signaling, primarily through the activation of cAMP-dependent protein kinase (PKA). PKA phosphorylates Gli proteins. In the case of Gli1 and 2 this stabilizes their association with SuFu and blocks their nuclear translocation [20], and in the case of Gli3 this results in its proteolysis into a transcriptional repressive form [70]. By these mechanisms, cAMP and PKA activity potently regulate expression of transcriptional targets of Shh [71]. The central role of cAMP/PKA in determining cellular response to Shh is illustrated by the localization of adenylate cyclase 3 and PKA to the base of the primary cilium between the basal position of the Gli transcription factors and the nucleus [72,73,74]. Importantly, deletion of PKA results in ligand-independent activation of Shh transcriptional targets [74]. Thus, cAMP levels can be viewed as regulating the coupling between Shh and expression of Shh target genes, and providing a mechanism for Shh-independent expression.

The role of cAMP in regulating Shh pathway activation has relevance to both normal cerebellar development and to the genesis of medulloblastoma. For example, the pituitary adenylate cyclase-activating polypeptide (PACAP) ligand and its GPCR PAC1 are expressed during neural development and act through Gs to block Shh-induced cell proliferation [75]. Moreover, targeted co-deletion of *PAC1* and *Ptch* increased the incidence of medulloblastoma compared to deletion of *Ptch* alone [75,76]. These data indicate that even in the setting of mutational activation of the Shh pathway, cAMP can exert control over the expression of Shh target genes. This is also indicated by a novel mouse model that identified Gα_s_ as a tumor suppressor in Shh subgroup medulloblastoma [77]. Knocking out expression of the Gα_s_ protein resulted in increased Shh signaling and decreased cAMP levels in GNPs and tumor cells.

The tumor phenotype of Gα_s_ null mice could be reversed using Rolipram, an inhibitor of cAMP-specific phosphodiesterases (PDE) belonging to the PDE4 family [77]. These and other PDEs have been shown to be altered in expression in medulloblastoma and other brain tumors [78] and Rolipram has been demonstrated to block brain tumor growth in several different models [78,79,80]. Rolipram and other specific PDE4 inhibitors have been extensively evaluated in clinical trials for depression, spinal cord injury, arthritis and COPD [78]. In general, specific PDE4 inhibitors have exhibited low toxicity, with nausea being the most common side effect reported for Rolipram. Therefore, a promising avenue for clinical research lies in optimizing strategies for cAMP elevation with these well-tolerated agents and the resulting activation of PKA.

## 10. Conclusions

**Figure 1:**
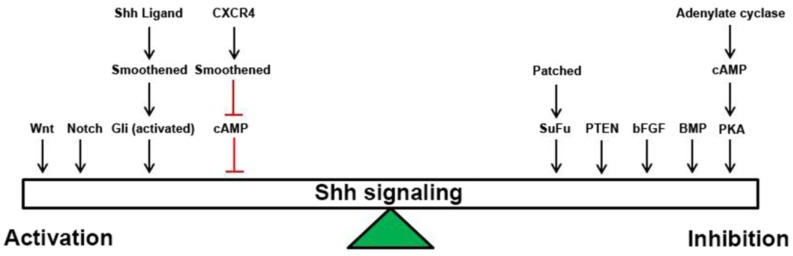
Graphical representation of the multiple factors and pathways that can tip the scales towards activation or inhibition of Shh response at the cellular level.

The multiplicity of pathways that modulate cellular responses to Shh up or down, suggests the presence of key integration points in cellular processing of Shh and convergent growth regulatory signals (Figure 1). We would propose that cAMP/PKA may be the critical parameter for determining whether a “Shh” signal is supra- or sub-threshold for the nuclear localization of Gli1/2 and transcriptional activation. Cyclic AMP is an attractive candidate for this regulatory role as its levels are acutely regulated through AC activity and its synthesis downstream of GPCRs, PDE activity and its degradation downstream of multiple signaling pathways. The fact that manipulation of cAMP levels can affect Shh target gene expression even in the absence of Shh ligand represents plasticity in cellular signaling and potentially mechanisms of resistance to Shh pathway antagonists. It follows that inhibition of Shh signaling at multiple levels in the pathway, focusing on regulators outside of the canonical ligand/receptor stage of signaling, will yield better anti-tumor activity and possibly fewer off-target effects. Compound screens have successfully identified compounds that activate Gs-associated GPCRs, resulting in inhibition of Shh signaling downstream of Gli1 [81,82] and antagonists of the Gi-coupled receptor CXCR4 have demonstrated efficacy in medulloblastoma models [64,83]. Inhibitors of PDEs exert potent anti-medulloblastoma effects *in vivo* [80,84] and cyclopamine treatment combined with drugs that elevate cAMP are more efficacious than either drug alone, even when used at lower doses [28]. Therefore, it is of the utmost importance to broaden our view of Shh responses and determine what other levels of signaling are important for tumor growth and targetable by pharmaceuticals. The clinical availability of well-tolerated PDE inhibitors and Shh pathway antagonists makes this approach particularly attractive.

## References

[1] Samkari A., White J.C., Packer R.J. (2015). Medulloblastoma: Toward biologically based management. Semin. Pediatr. Neurol..

[2] Louis D.N., Ohgaki H., Wiestler O., Cavenee W.K. (2007). WHO Classification of Tumours of the Central Nervous System.

[3] Northcott P.A., Korshunov A., Witt H., Hielscher T., Eberhart C.G., Mack S., Bouffet E., Clifford S.C., Hawkins C.E., French P. (2011). Medulloblastoma comprises four distinct molecular variants. J. Clin. Oncol..

[4] Kool M., Korshunov A., Remke M., Jones D.T., Schlanstein M., Northcott P.A., Cho Y.J., Koster J., Schouten-Van Meeteren A., van Vuurden D. (2012). Molecular subgroups of medulloblastoma: an international meta-analysis of transcriptome, genetic aberrations, and clinical data of WNT, SHH, Group 3, and Group 4 medulloblastomas. Acta Neuropathol..

[5] Hallahan A.R., Pritchard J.I., Hansen S., Benson M., Stoeck J., Hatton B.A., Russell T.L., Ellenbogen R.G., Bernstein I.D., Beachy P.A. (2004). The SmoA1 mouse model reveals that notch signaling is critical for the growth and survival of sonic hedgehog-induced medulloblastomas. Cancer Res..

[6] Hatton B.A., Villavicencio E.H., Tsuchiya K.D., Pritchard J.I., Ditzler S., Pullar B., Hansen S., Knoblaugh S.E., Lee D., Eberhart C.G. (2008). The Smo/Smo model: Hedgehog-induced medulloblastoma with 90% incidence and leptomeningeal spread. Cancer Res..

[7] Goodrich L.V., Milenkovic L., Higgins K.M., Scott M.P. (1997). Altered neural cell fates and medulloblastoma in mouse patched mutants. Science.

[8] Vaillant C., Monard D. (2009). SHH pathway and cerebellar development. Cerebellum.

[9] Klein R.S., Rubin J.B., Gibson H.D., DeHaan E.N., Alvarez-Hernandez X., Segal R.A., Luster A.D. (2001). SDF-1 alpha induces chemotaxis and enhances Sonic hedgehog-induced proliferation of cerebellar granule cells. Development.

[10] Borghesani P.R., Peyrin J.M., Klein R., Rubin J., Carter A.R., Schwartz P.M., Luster A., Corfas G., Segal R.A. (2002). BDNF stimulates migration of cerebellar granule cells. Development.

[11] Johnson R.L., Rothman A.L., Xie J., Goodrich L.V., Bare J.W., Bonifas J.M., Quinn A.G., Myers R.M., Cox D.R., Epstein E.H. (1996). Human homolog of patched, a candidate gene for the basal cell nevus syndrome. Science.

[12] Taylor M.D., Northcott P.A., Korshunov A., Remke M., Cho Y.J., Clifford S.C., Eberhart C.G., Parsons D.W., Rutkowski S., Gajjar A. (2012). Molecular subgroups of medulloblastoma: The current consensus. Acta Neuropathol..

[13] Robarge K.D., Brunton S.A., Castanedo G.M., Cui Y., Dina M.S., Goldsmith R., Gould S.E., Guichert O., Gunzner J.L., Halladay J. (2009). GDC-0449-a potent inhibitor of the hedgehog pathway. Bioorg. Med. Chem. Lett..

[14] Pan S., Wu X., Jiang J., Gao W., Wan Y., Cheng D., Han D., Liu J., Englund N.P., Wang Y. (2010). Discovery of NVP-LDE225, a potent and selective smoothened antagonist. ACS Med. Chem. Lett..

[15] Rudin C.M. (2012). Vismodegib. Clin. Cancer Res..

[16] Rudin C.M., Hann C.L., Laterra J., Yauch R.L., Callahan C.A., Fu L., Holcomb T., Stinson J., Gould S.E., Coleman B. (2009). Treatment of Medulloblastoma with Hedgehog Pathway Inhibitor GDC-0449. N. Engl. J. Med..

[17] Yauch R.L., Dijkgraaf G.J., Alicke B., Januario T., Ahn C.P., Holcomb T., Pujara K., Stinson J., Callahan C.A., Tang T. (2009). Smoothened mutation confers resistance to a Hedgehog pathway inhibitor in medulloblastoma. Science.

[18] Choudhry Z., Rikani A.A., Choudhry A.M., Tariq S., Zakaria F., Asghar M.W., Sarfraz M.K., Haider K., Shafiq A.A., Mobassarah N.J. (2014). Sonic hedgehog signalling pathway: A complex network. Ann. Neurosci..

[19] Riobo N.A., Lu K., Ai X., Haines G.M., Emerson C.P. (2006). Phosphoinositide 3-kinase and Akt are essential for Sonic Hedgehog signaling. Proc. Natl. Acad. Sci. USA.

[20] Pan Y., Bai C.B., Joyner A.L., Wang B. (2006). Sonic hedgehog signaling regulates Gli2 transcriptional activity by suppressing its processing and degradation. Mol. Cell. Biol..

[21] Katoh Y., Katoh M. (2009). Hedgehog target genes: mechanisms of carcinogenesis induced by aberrant hedgehog signaling activation. Curr. Mol. Med..

[22] Chandra V., Das T., Gulati P., Biswas N.K., Rote S., Chatterjee U., Ghosh S.N., Deb S., Saha S.K., Chowdhury A.K. (2015). Hedgehog signaling pathway is active in GBM with GLI1 mRNA expression showing a single continuous distribution rather than discrete high/low clusters. PLoS ONE.

[23] Bumcrot D.A., Takada R., McMahon A.P. (1995). Proteolytic processing yields two secreted forms of sonic hedgehog. Mol. Cell. Biol..

[24] Pepinsky R.B., Zeng C., Wen D., Rayhorn P., Baker D.P., Williams K.P., Bixler S.A., Ambrose C.M., Garber E.A., Miatkowski K. (1998). Identification of a palmitic acid-modified form of human Sonic hedgehog. J. Biol. Chem..

[25] Vyas N., Goswami D., Manonmani A., Sharma P., Ranganath H.A., VijayRaghavan K., Shashidhara L.S., Sowdhamini R., Mayor S. (2008). Nanoscale organization of hedgehog is essential for long-range signaling. Cell.

[26] Jakobs P., Exner S., Schurmann S., Pickhinke U., Bandari S., Ortmann C., Kupich S., Schulz P., Hansen U., Seidler D.G. (2014). Scube2 enhances proteolytic Shh processing from the surface of Shh-producing cells. J. Cell. Sci..

[27] Hadden M.K. (2013). Hedgehog pathway inhibitors: A patent review (2009–present). Expert Opin. Ther. Pat..

[28] Powers G.L., Hammer K.D., Domenech M., Frantskevich K., Malinowski R.L., Bushman W., Beebe D.J., Marker P.C. (2015). Phosphodiesterase 4D inhibitors limit prostate cancer growth potential. Mol. Cancer Res..

[29] Logan C.Y., Nusse R. (2004). The Wnt signaling pathway in development and disease. Annu. Rev. Cell Dev. Biol..

[30] Rao T.P., Kuhl M. (2010). An updated overview on Wnt signaling pathways: A prelude for more. Circ. Res..

[31] Nusse R. Wnt/beta-catenin signaling target genes. http://web.stanford.edu/~rnusse/pathways/targets.html.

[32] Teodorczyk M., Schmidt M.H. (2014). Notching on cancer’s door: Notch signaling in brain tumors. Front. Oncol..

[33] Gaiano N., Nye J.S., Fishell G. (2000). Radial glial identity is promoted by Notch1 signaling in the murine forebrain. Neuron.

[34] Chiba S. (2006). Notch signaling in stem cell systems. Stem Cells.

[35] Solecki D.J., Liu X.L., Tomoda T., Fang Y., Hatten M.E. (2001). Activated Notch2 signaling inhibits differentiation of cerebellar granule neuron precursors by maintaining proliferation. Neuron.

[36] Fan X., Mikolaenko I., Elhassan I., Ni X., Wang Y., Ball D., Brat D.J., Perry A., Eberhart C.G. (2004). Notch1 and Notch2 Have Opposite Effects on Embryonal Brain Tumor Growth. Cancer Res..

[37] Cordeiro B.M., Oliveira I.D., Alves M.T., Saba-Silva N., Capellano A.M., Cavalheiro S., Dastoli P., Toledo S.R. (2014). SHH, WNT, and NOTCH pathways in medulloblastoma: When cancer stem cells maintain self-renewal and differentiation properties. Childs Nerv. Syst..

[38] Fiaschetti G., Schroeder C., Castelletti D., Arcaro A., Westermann F., Baumgartner M., Shalaby T., Grotzer M.A. (2014). NOTCH ligands JAG1 and JAG2 as critical pro-survival factors in childhood medulloblastoma. Acta Neuropathol. Commun..

[39] De Antonellis P., Medaglia C., Cusanelli E., Andolfo I., Liguori L., de Vita G., Carotenuto M., Bello A., Formiggini F., Galeone A. (2011). MiR-34a targeting of Notch ligand delta-like 1 impairs CD15+/CD133+ tumor-propagating cells and supports neural differentiation in medulloblastoma. PLoS ONE.

[40] Ingram W.J., McCue K.I., Tran T.H., Hallahan A.R., Wainwright B.J. (2008). Sonic Hedgehog regulates Hes1 through a novel mechanism that is independent of canonical Notch pathway signalling. Oncogene.

[41] Dave R.K., Ellis T., Toumpas M.C., Robson J.P., Julian E., Adolphe C., Bartlett P.F., Cooper H.M., Reynolds B.A., Wainwright B.J. (2011). Sonic hedgehog and notch signaling can cooperate to regulate neurogenic divisions of neocortical progenitors. PLoS ONE.

[42] Natarajan S., Li Y., Miller E.E., Shih D.J., Taylor M.D., Stearns T.M., Bronson R.T., Ackerman S.L., Yoon J.K., Yun K. (2013). Notch1-Induced Brain Tumor Models the Sonic Hedgehog Subgroup of Human Medulloblastoma. Cancer Res..

[43] Hatton B.A., Villavicencio E.H., Pritchard J., LeBlanc M., Hansen S., Ulrich M., Ditzler S., Pullar B., Stroud M.R., Olson J.M. (2010). Notch signaling is not essential in sonic hedgehog-activated medulloblastoma. Oncogene.

[44] Julian E., Dave R.K., Robson J.P., Hallahan A.R., Wainwright B.J. (2010). Canonical Notch signaling is not required for the growth of Hedgehog pathway-induced medulloblastoma. Oncogene.

[45] Grimmer M.R., Weiss W.A. (2008). BMPs oppose Math1 in cerebellar development and in medulloblastoma. Genes Dev..

[46] Shi Y., Massague J. (2003). Mechanisms of TGF-beta signaling from cell membrane to the nucleus. Cell.

[47] Alder J., Lee K.J., Jessell T.M., Hatten M.E. (1999). Generation of cerebellar granule neurons *in vivo* by transplantation of BMP-treated neural progenitor cells. Nat. Neurosci..

[48] Rios I., Alvarez-Rodriguez R., Marti E., Pons S. (2004). Bmp2 antagonizes sonic hedgehog-mediated proliferation of cerebellar granule neurones through Smad5 signalling. Development.

[49] Zhao H., Ayrault O., Zindy F., Kim J.H., Roussel M.F. (2008). Post-transcriptional down-regulation of Atoh1/Math1 by bone morphogenic proteins suppresses medulloblastoma development. Genes Dev..

[50] Hallahan A.R., Pritchard J.I., Chandraratna R.A., Ellenbogen R.G., Geyer J.R., Overland R.P., Strand A.D., Tapscott S.J., Olson J.M. (2003). BMP-2 mediates retinoid-induced apoptosis in medulloblastoma cells through a paracrine effect. Nat. Med..

[51] Wechsler-Reya R.J., Scott M.P. (1999). Control of neuronal precursor proliferation in the cerebellum by Sonic Hedgehog. Neuron.

[52] Fogarty M.P., Emmenegger B.A., Grasfeder L.L., Oliver T.G., Wechsler-Reya R.J. (2007). Fibroblast growth factor blocks Sonic hedgehog signaling in neuronal precursors and tumor cells. Proc. Natl. Acad. Sci. USA.

[53] Emmenegger B.A., Hwang E.I., Moore C., Markant S.L., Brun S.N., Dutton J.W., Read T.A., Fogarty M.P., Singh A.R., Durden D.L. (2013). Distinct roles for fibroblast growth factor signaling in cerebellar development and medulloblastoma. Oncogene.

[54] Steck P.A., Pershouse M.A., Jasser S.A., Yung W.K., Lin H., Ligon A.H., Langford L.A., Baumgard M.L., Hattier T., Davis T. (1997). Identification of a candidate tumour suppressor gene, MMAC1, at chromosome 10q23.3 that is mutated in multiple advanced cancers. Nat. Genet..

[55] Li J., Yen C., Liaw D., Podsypanina K., Bose S., Wang S.I., Puc J., Miliaresis C., Rodgers L., McCombie R. (1997). PTEN, a putative protein tyrosine phosphatase gene mutated in human brain, breast, and prostate cancer. Science.

[56] Castellino R.C., Durden D.L. (2007). Mechanisms of disease: the PI3K-Akt-PTEN signaling node—An intercept point for the control of angiogenesis in brain tumors. Nat. Clin. Pract. Neurol..

[57] Griffin C.A., Hawkins A.L., Packer R.J., Rorke L.B., Emanuel B.S. (1988). Chromosome abnormalities in pediatric brain tumors. Cancer Res..

[58] Lastowska M., Al-Afghani H., Al-Balool H.H., Sheth H., Mercer E., Coxhead J.M., Redfern C.P., Peters H., Burt A.D., Santibanez-Koref M. (2013). Identification of a neuronal transcription factor network involved in medulloblastoma development. Communication.

[59] Metcalfe C., Alicke B., Crow A., Lamoureux M., Dijkgraaf G.J., Peale F., Gould S.E., de Sauvage F.J. (2013). PTEN loss mitigates the response of medulloblastoma to Hedgehog pathway inhibition. Cancer Res..

[60] Castellino R.C., Barwick B.G., Schniederjan M., Buss M.C., Becher O., Hambardzumyan D., Macdonald T.J., Brat D.J., Durden D.L. (2010). Heterozygosity for Pten promotes tumorigenesis in a mouse model of medulloblastoma. PLoS ONE.

[61] Hartmann W., Digon-Sontgerath B., Koch A., Waha A., Endl E., Dani I., Denkhaus D., Goodyer C.G., Sorensen N., Wiestler O.D. (2006). Phosphatidylinositol 3′-kinase/AKT signaling is activated in medulloblastoma cell proliferation and is associated with reduced expression of PTEN. Clin. Cancer Res..

[62] Dorsam R.T., Gutkind J.S. (2007). G-protein-coupled receptors and cancer. Nat. Rev. Cancer.

[63] Tautermann C.S. (2014). GPCR structures in drug design, emerging opportunities with new structures. Bioorg. Med. Chem. Lett..

[64] Sengupta R., Dubuc A., Ward S., Yang L., Northcott P., Woerner B.M., Kroll K., Luo J., Taylor M.D., Wechsler-Reya R.J. (2012). CXCR4 activation defines a new subgroup of Sonic hedgehog-driven medulloblastoma. Cancer Res..

[65] Guo F., Wang Y., Liu J., Mok S.C., Xue F., Zhang W. (2015). CXCL12/CXCR4: A symbiotic bridge linking cancer cells and their stromal neighbors in oncogenic communication networks. Oncogene.

[66] Tissir F., Wang C.E., Goffinet A.M. (2004). Expression of the chemokine receptor Cxcr4 mRNA during mouse brain development. Brain Res. Dev. Brain Res..

[67] Hagihara K., Zhang E.E., Ke Y.H., Liu G., Liu J.J., Rao Y., Feng G.S. (2009). Shp2 acts downstream of SDF-1alpha/CXCR4 in guiding granule cell migration during cerebellar development. Dev. Biol..

[68] Hurowitz E.H., Melnyk J.M., Chen Y.J., Kouros-Mehr H., Simon M.I., Shizuya H. (2000). Genomic characterization of the human heterotrimeric G protein alpha, beta, and gamma subunit genes. DNA Res..

[69] Neves S.R., Ram P.T., Iyengar R. (2002). G protein pathways. Science.

[70] Pal K., Mukhopadhyay S. (2015). Primary cilium and sonic hedgehog signaling during neural tube patterning: Role of GPCRs and second messengers. Dev. Neurobiol..

[71] Makinodan E., Marneros A.G. (2012). Protein kinase A activation inhibits oncogenic Sonic hedgehog signalling and suppresses basal cell carcinoma of the skin. Exp. Dermatol..

[72] Wang Z., Phan T., Storm D.R. (2011). The type 3 adenylyl cyclase is required for novel object learning and extinction of contextual memory: Role of cAMP signaling in primary cilia. J. Neurosci..

[73] Miyoshi K., Kasahara K., Miyazaki I., Asanuma M. (2009). Lithium treatment elongates primary cilia in the mouse brain and in cultured cells. Biochem. Biophys. Res. Commun..

[74] Tuson M., He M., Anderson K.V. (2011). Protein kinase A acts at the basal body of the primary cilium to prevent Gli2 activation and ventralization of the mouse neural tube. Development.

[75] Nicot A., Lelievre V., Tam J., Waschek J.A., DiCicco-Bloom E. (2002). Pituitary adenylate cyclase-activating polypeptide and sonic hedgehog interact to control cerebellar granule precursor cell proliferation. J. Neurosci..

[76] Niewiadomski P., Zhujiang A., Youssef M., Waschek J.A. (2013). Interaction of PACAP with Sonic hedgehog reveals complex regulation of the hedgehog pathway by PKA. Cell. Signal..

[77] He X., Zhang L., Chen Y., Remke M., Shih D., Lu F., Wang H., Deng Y., Yu Y., Xia Y. (2014). The G protein alpha subunit Galphas is a tumor suppressor in Sonic hedgehog-driven medulloblastoma. Nat. Med..

[78] Sengupta R., Sun T., Warrington N.M., Rubin J.B. (2011). Treating brain tumors with PDE4 inhibitors. Trends Pharmacol. Sci..

[79] Warrington N.M., Gianino S.M., Jackson E., Goldhoff P., Garbow J.R., Piwnica-Worms D., Gutmann D.H., Rubin J.B. (2010). Cyclic AMP suppression is sufficient to induce gliomagenesis in a mouse model of neurofibromatosis-1. Cancer Res..

[80] Goldhoff P., Warrington N.M., Limbrick D.D., Hope A., Woerner B.M., Jackson E., Perry A., Piwnica-Worms D., Rubin J.B. (2008). Targeted inhibition of cyclic AMP phosphodiesterase-4 promotes brain tumor regression. Clin. Cancer Res..

[81] Bassilana F., Carlson A., DaSilva J.A., Grosshans B., Vidal S., Beck V., Wilmeringwetter B., Llamas L.A., Showalter T.B., Rigollier P. (2014). Target identification for a Hedgehog pathway inhibitor reveals the receptor GPR39. Nat. Chem. Biol..

[82] Schmidt A.L., de Farias C.B., Abujamra A.L., Kapczinski F., Schwartsmann G., Brunetto A.L., Roesler R. (2010). BDNF and PDE4, but not the GRPR, regulate viability of human medulloblastoma cells. J. Mol. Neurosci..

[83] Rubin J.B., Kung A.L., Klein R.S., Chan J.A., Sun Y., Schmidt K., Kieran M.W., Luster A.D., Segal R.A. (2003). A small-molecule antagonist of CXCR4 inhibits intracranial growth of primary brain tumors. Proc. Natl. Acad. Sci. USA.

[84] Yang L., Jackson E., Woerner B.M., Perry A., Piwnica-Worms D., Rubin J.B. (2007). Blocking CXCR4-mediated cyclic AMP suppression inhibits brain tumor growth *in vivo*. Cancer Res..

